# Enhanced Understanding of Infectious Diseases by Fusing Multiple Datasets: A Case Study on Malaria in the Western Brazilian Amazon Region

**DOI:** 10.1371/journal.pone.0027462

**Published:** 2011-11-08

**Authors:** Denis Valle, James S. Clark, Kaiguang Zhao

**Affiliations:** 1 University Program in Ecology, Duke University, Durham, North Carolina, United States of America; 2 Department of Biology, Duke University, Durham, North Carolina, United States of America; 3 Nicholas School of the Environment, Duke University, Durham, North Carolina, United States of America; Asociacion Civil Impacta Salud y Educacion, Peru

## Abstract

**Background:**

A common challenge to the study of several infectious diseases consists in combining limited cross-sectional survey data, collected with a more sensitive detection method, with a more extensive (but biased) syndromic sentinel surveillance data, collected with a less sensitive method. Our article describes a novel modeling framework that overcomes this challenge, resulting in enhanced understanding of malaria in the Western Brazilian Amazon.

**Methodology/Principal Findings:**

A cohort of 486 individuals was monitored using four cross-sectional surveys, where all participants were sampled regardless of symptoms (aggressive-active case detection), resulting in 1,383 microscopy and 1,400 polymerase chain reaction tests. Data on the same individuals were also obtained from the local surveillance facility (i.e., passive and active case detection), totaling 1,694 microscopy tests. Our model accommodates these multiple pathogen and case detection methods. This model is shown to outperform logistic regression in terms of interpretability of its parameters, ability to recover the true parameter values, and predictive performance. We reveal that the main infection determinant was the extent of forest, particularly during the rainy season and in close proximity to water bodies, and participation on forest activities. We find that time residing in Acrelandia (as a proxy for past malaria exposure) decreases infection risk but surprisingly increases the likelihood of reporting symptoms once infected, possibly because non-naïve settlers are only susceptible to more virulent *Plasmodium* strains. We suggest that the search for asymptomatic carriers should focus on those at greater risk of being infected but lower risk of reporting symptoms once infected.

**Conclusions/Significance:**

The modeling framework presented here combines cross-sectional survey data and syndromic sentinel surveillance data to shed light on several aspects of malaria that are critical for public health policy. This framework can be adapted to enhance inference on infectious diseases whenever asymptomatic carriers are important and multiple datasets are available.

## Introduction

Extensive syndromic sentinel surveillance data are often routinely collected by public health agencies. However, estimates of disease prevalence based on these data are known to be biased because only symptomatic individuals are sampled [1], [2]. Furthermore, because of the sentinel surveillance network extent, cheaper and less sensitive diagnostic methods are typically employed. Researchers also collect data to study infectious disease risk factors and asymptomatic pathogen carriers, but using cross-sectional surveys and more expensive and sensitive diagnostic methods. These data, however, are often geographically and temporally limited and thus are not as abundant as sentinel surveillance data. Robust inference on disease prevalence and risk factors would ideally combine these datasets because they clearly complement each other; unfortunately, standard statistical tools are not well suited for this task. We describe here a novel statistical model that coherently combines these disparate datasets, allowing for enhanced inference on infectious diseases.

Our study focuses on malaria. Malaria is responsible for ∼3% of the total global disease burden [3], affecting approximately half of the world's population [4] and significantly hindering economic and social development of tropical countries [5]. Despite its public health relevance and recent increased attention to malaria research and control [6], malaria risk factors remain difficult to evaluate, due both to the idiosyncrasies of how data are collected (as detailed below) and the fact that not all infected individuals are symptomatic. Our approach addresses these challenges, providing sharper inference on *Plasmodium* infection risk factors, factors determining symptom status given infection, and overall infection and disease prevalence. We first describe the statistical model, then we compare its performance against standard logistic regression using simulated and real data, and finally we apply it to a large malaria dataset collected in the Western Brazilian Amazon.

In Brasil, malaria cases are concentrated in the Amazon region [7], resulting in substantial morbidity [8], [9]. Similar to other countries (e.g., India, [10]), the malaria surveillance data from the Brazilian government consist of microscopy results from predominantly symptomatic individuals, sampled through active and passive case detection (ACD and PCD, respectively). ACD data are obtained by health agents during home visits to symptomatic individuals whereas PCD data come from health facilities, visited by individuals who believe they have malaria [11]. Inherent biases in both datasets make it difficult to determine overall malaria prevalence and the factors that influence it [1], [12]. Aggressive active case detection (AACD) has been proposed as an alternative surveillance technique, consisting of cross-sectional surveys where all individuals are sampled, regardless of symptom status [11]. AACD data can be used to estimate infection prevalence and its determinants and the size of the reservoir represented by asymptomatic *Plasmodium* carriers [12]–[14]. Drawbacks of AACD include high costs and the often low acceptability from the population [12], [14], which often limits AACD data to a short time-frame and a small geographical area. As a consequence, AACD data might not be as well suited as ACD/PCD data in determining the effect of covariates that change substantially in time and/or space (e.g., precipitation and presence of wetlands).

Imperfect *Plasmodium* detection is a concern for all surveillance methods. The Brazilian Health Ministry primarily makes use of microscopy of thick blood smears, because it is relatively inexpensive and straight-forward [15]. However, microscopy has limited ability to detect the pathogen when parasitemia is low [16]–[18]. In research settings, Polymerase Chain Reaction (PCR) has been extensively used as the standard against which the sensitivity and specificity of other detection methods (e.g., microscopy and rapid diagnostic tests) are evaluated. Unfortunately, PCR data is often not available due to costs and expertise required for the procedure [19], [20].

How does one integrate the less biased but more limited dataset (e.g., data from AACD) with a more extensive, time continuous and biased dataset (e.g., data from ACD/PCD)? Furthermore, how can the more sensitive but limited PCR dataset be used jointly with the less sensitive but more extensive microscopy dataset? Logistic regression is the most common statistical tool used to analyze individual-level disease data. However, logistic regression does not correct for the biases in the ACD/PCD dataset, even if dummy covariates are added to represent differences in how individuals were sampled (e.g., AACD, ACD, and PCD). It also does not accommodate detection error rates for the different *Plasmodium* detection methods. In recognition of these problems, analysis might focus on the most sensitive pathogen detection method (i.e., PCR) and less biased case detection method (i.e., AACD), with the drawback of ignoring considerable information contained in the rest of the data.

Logistic regression also does not allow for important conditional relationships that determine malaria risk. Malaria researchers typically assume perfect detection and choose to model either the probability of being diseased (i.e., 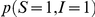) or the probability of being infected (i.e., 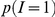), where *S* and *I* stand for symptom and infection status. These probabilities are related and models can be developed to combine them in a statistically and biologically coherent way. Our model factors 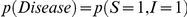 as 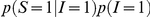, allowing us to separately evaluate infection risk factors (i.e., 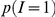) from risk factors of symptoms given infection (i.e., 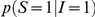). This approach can provide inference on factors that influence the joint distribution of symptom and infection statuses. For example, we can coherently estimate the prevalence of asymptomatic carriers, namely 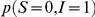, and the factors that influence it. The limitations of standard statistical tools prompted us to create a customized method to analyze our data.

Here, we illustrate how inference on malaria risk factors and infection/disease prevalence can be improved using a hierarchical framework based on the joint distribution of symptom and infections statuses and by properly accommodating the different pathogen and case detection methods. First, we detail the model. Then, we compare the performance of this method to that of typical logistic regressions using simulated and real data. Finally, we apply this model on a large malaria dataset collected in the Western Brazilian Amazon and discuss the implication of our findings.

## Methods

### Ethics Statement

The study protocol was approved by the Ethical Review Board of the Institute of Biomedical Sciences of the University of São Paulo, Brazil (318/2002 and 538/2004) and we obtained written informed consent from each adult participant and from the parent or legal guardian of every minor.

### Data

Data were collected in a rural settlement area, in a region known as Ramal Granada (Acrelandia, Acre, Brasil), on 486 individuals that agreed to participate in the study. AACD data come from four cross-sectional surveys (March/April 2004, September/October 2004, February/March 2005, and October/November 2006) in which all study participants that were present at the time of the survey were sampled, regardless of their symptomatic status. This dataset contained a total of 1383 microscopy and 1400 PCR malaria tests. Further details on the area, data collection, and characteristics of this cohort can be found elsewhere [11], [21], [22]. We gathered ACD/PCD data by searching the malaria records at the local health facility. All malaria records between 2004 and 2007 from the AACD study participants were entered in a database, resulting in a total of 1694 microscopy tests, with approximately 94% of the individuals feeling symptomatic when tested.

### Model Description

We start by describing some basic conditional probabilities for our model and their associated assumptions. We then proceed to detail the likelihood associated with each potential outcome. We conclude this section with a description of how we fit the model.

#### Plasmodium detection

We consider data from two *Plasmodium* detection methods, namely microscopy and polymerase chain reaction (PCR). Let 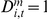 stand for a positive *Plasmodium* detection using microscopy for individual *i* at time *t*. Let 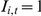 and 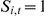 stand for being infected and having malaria symptoms, respectively. Note that 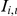 is a latent variable because we never directly observe it. Using these definitions, let 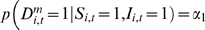
and 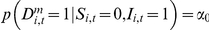 be the microscopy sensitivity given that 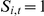 and 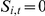, respectively. We allow sensitivity to depend on symptom status because it has been shown that low-grade infections (i.e., low density of parasites in the blood) are associated with asymptomatic cases and failure to detect them with microscopy [16]–[18], [23]. Furthermore, let 
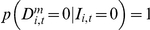
 be the microscopy specificity. We set the specificity of the microscopy to one because it is virtually impossible for an experienced microscopist to identify malaria pathogens on a blood sample from an uninfected patient, regardless of the symptomatic status of the patient (Ferreira, personal communication; [17]).

In relation to PCR, let 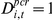 stand for a positive *Plasmodium* detection using PCR for individual *i* at time *t*. Let the PCR sensitivity and specificity be denoted by 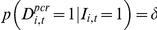 and 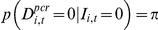, respectively. Errors in amplification or contamination of the sample can produce both false-positives and false-negatives [17]. From prior knowledge, we know that the sensitivity of PCR is greater than that of microscopy and that microscopy sensitivity is probably greater when the individual is symptomatic than when not symptomatic (i.e., 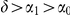) [20]. Finally, we assume that PCR sensitivity and specificity are not influenced by microscopy detection and symptomatic status of the individual, given infection status. The assumption of conditional independence between PCR and microscopy results seems reasonable because detections are based on fundamentally different biological processes [24], [25]. We adopted uniform priors for the sensitivity and specificity of PCR, where the limits were based on earlier reports on PCR error rates [26], [27]. More specifically, the joint prior adopted for these detection parameters was a uniform distribution in the set 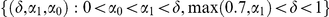.

#### Infection risk

We are primarily interested in the probability that individual *i* at time *t* is infected with *Plasmodium* (i.e., 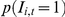) and the associated risk factors. We assume that this probability is given by
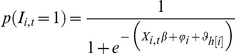



where 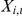 is the design vector and 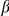 is the vector with the corresponding parameters. The design vector 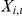 contains potential risk factors. For our case study using data from the Western Brazilian Amazon, these covariates were gender, educational level, age, time in Acrelandia (as a proxy for past exposure to malaria), if participates on extractivism activities, if hunts or fishes, if works as chain sawyer, if shares the house with somebody that had a positive malaria diagnosis in the past 30 days, surface water area, forest area, deforestation rate, precipitation, and a drought index. These covariates are detailed in Appendix S1. Individual and household-level random effects are denoted by 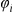 and 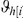, respectively, where *h[i]* indexes the household where the *i^th^* person resides. These random effects were modeled as 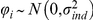 and 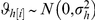, where 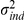 and 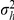 are the individual and household-level random effect variances, respectively.

#### Symptomatic status

We assume that the probability of being symptomatic given that the person is infected is given by
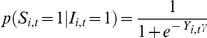



where 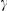 is a vector of parameters to be estimated and 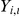 is the design vector. We assume that the covariates most likely to influence this probability are variables related to the individual's immune system and not variables related to present exposure to vectors. Thus, for our Western Brazilian Amazon case study, the covariates in 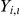 were age, gender, and time in Acrelandia (as a proxy for past malaria exposure). Finally, we assumed that the probability of having symptoms despite not being infected 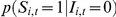 was a constant parameter to be estimated.

#### Likelihood

**Figure 1:**
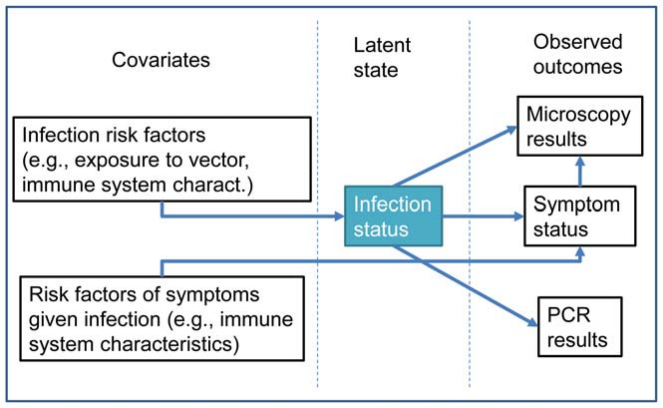
Graphical representation of the proposed model, illustrating some of the modeled conditional relationships.

The definitions above are the basis for the hierarchical model that we built (depicted in Fig. 1), borrowing some ideas from Clark & Hersh [28]. These definitions and model structure allow us to describe the likelihoods of all the possible outcomes in AACD (Table S1). For the ACD and PCD datasets, we start by noting that 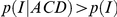 and 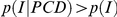, because ACD and PCD focuses mostly on symptomatic individuals. Therefore, we can assume that knowing whether the person was sampled in ACD or PCD does not bring any additional information about the risk of being infected if we condition on symptomatic status. More formally, we assume that 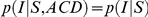 and 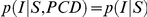. Based on these assumptions, it can be shown that the likelihood for each outcome will be similar to those for AACD with the exception that it will have a correction term of the form 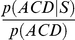 or 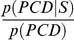. Here, 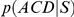 and 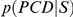 are the conditional probability that an individual with symptom status *S* is sampled through ACD or PCD, respectively, and 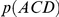 and 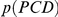 are the corresponding marginal probabilities. The likelihood of all the possible outcomes in ACD and PCD is shown in Table S2. The detailed derivation of these likelihoods is given in Appendix S2.

An important assumption in our analysis of the ACD/PCD dataset is that malaria tests (and the symptomatic status at the time of the test) more than one week apart from each other were considered to be independent. There were some cases where symptomatic individuals would choose to be tested multiple times within a short period of time (<7 days), probably expecting a positive result or the symptoms to ameliorate. To avoid making several assumptions regarding the temporal dependencies of symptoms and test results from these multiple tests, we chose to retain just the first test and the associated symptomatic status whenever we detected multiple tests within this short time-frame.

#### Full model

Let 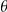 be all the parameters we will estimate and let 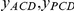 and 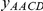 be the different datasets, where subscripts denote how individuals were sampled. Assuming conditional independence given the parameters 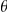, the full model can be written as
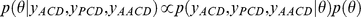


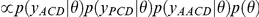



where 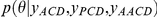 is the posterior distribution of the parameters to be estimated, 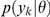 is the likelihood of dataset *k* (Table S1 and Table S2) and 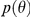 are the priors. All the estimated parameters 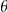 are listed and described in Table 1, together with their associated priors.

**Table 1:** List of all the estimated parameters and the associated priors.

Parameter	Description	Prior
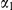	Microscopy sensitivity given S = 1	uniform in the set 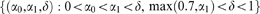
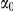	Microscopy sensitivity given S = 0	
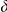	PCR sensitivity	
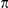	PCR specificity	Unif(0.97,1)
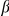	Covariates of infection risk factors	Unif(−10,10)
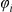	Individual level random effects	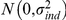
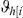	Household level random effects	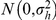
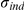	Standard deviation of the individual-level random effects	Unif(0,100)
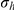	Standard deviation of the household-level random effects	Unif(0,100)
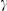	Covariates of risk factors of symptoms given infection	Unif(−10,10)
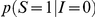	Probability of symptoms given no infection	Unif(0,1)
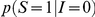	Probability of being sampled through PCD given no symptoms	uniform in the set 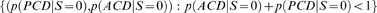
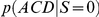	Probability of being sampled through ACD given no symptoms	
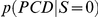	Probability of being sampled through PCD given symptoms	uniform in the set 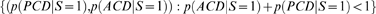
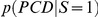	Probability of being sampled through ACD given symptoms	

This model was fitted using a Gibbs sampler. Most parameters were updated using a Metropolis sampling step and the few parameters that were updated via a Gibbs sampling step have their full conditional distributions described in Appendix S3. In total, 150,000 iterations were run and the initial 20,000 iterations were discarded as burn-in. Convergence was assessed using trace-plots of the parameters.

### Model Performance

We compare the proposed model with standard logistic regressions, both with and without individual and household level random effects. Let 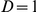 be a positive *Plasmodium* detection, either from microscopy, PCR or both. The response variable for these logistic regressions were proxies for a) disease: a person having symptoms and a positive detection (i.e., 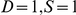); and b) infection: a person having a positive detection (i.e., 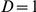) (Table 2). To mimic how researchers would typically use these multiple datasets (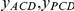 and 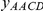), we merged the three datasets into a single one and added two dummy covariates in the logistic regressions to allow for differences between datasets.

**Table 2:** Description of all the modeling approaches employed in the simulation and validation exercises.

Models	Outcome	Description	Random effects
1	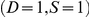 , 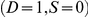 , 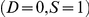 , 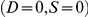	proposed model	Yes
2†	Disease 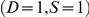	logistic regression	No
3†	Infection 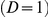	logistic regression	No
4††	Disease 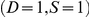	logistic regression	Yes
5††	Infection 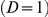	logistic regression	Yes
No covariate	Disease 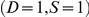 Infection 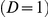	Uses the proportion of 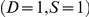 and 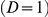 in the training dataset to predict outcomes for the validation dataset	No

† these models were fit using the ‘glm’ function in R.

†† these models were fit using the ‘lmer’ function in R.

These different statistical methodologies were compared using both simulated and real data. Simulated data were used to compare the different methods in relation to how well they retrieved the true parameters influencing infection probability. To evaluate the importance of combining these multiple datasets, we further compared how inference from the proposed model would change if fitted only to the PCR dataset versus all datasets. Details of how the simulated data were generated are given in Appendix S4, Table S3, and in Table S4. We also compared how well each model predicted the real data, using a 10-fold cross validation. This validation exercise consisted in fitting these models to 90% of the real data and comparing their predictions for the remaining 10%. This was done ten times with different portions of the data retained for validation at each time. Each method predicted which individuals had a positive test result (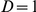) and which individuals had a positive test result and were symptomatic (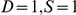). We summarized this information as a) the proportion of individuals correctly predicted as 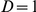 or 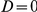; and b) the proportion of individuals correctly predicted as 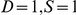 or not 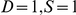. For this validation exercise, we also evaluated the predictive ability of the chosen covariates by adding the prediction results from a model that simply used the proportion of individuals with 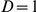 (or 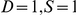) in the training dataset. All statistical procedures and graphics were performed in R [29].

## Results

### Model performance

**Figure 2:**
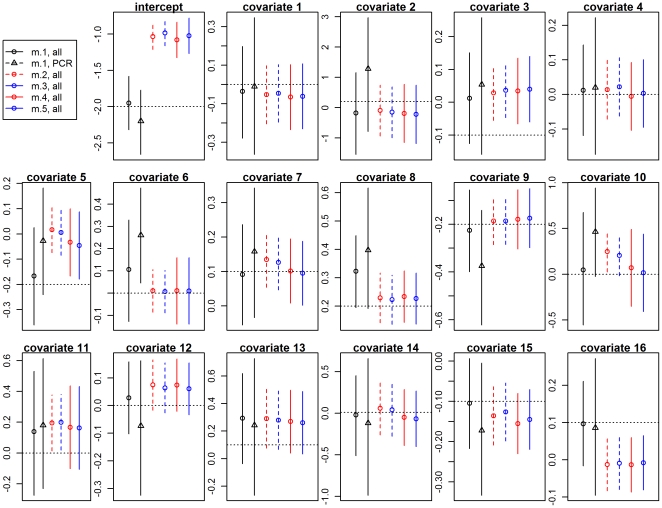
Comparison of models using simulated data. The true values of the infection risk factor parameters are depicted in horizontal black dashed lines. Logistic regression models with disease (models 2 and 4) and infection (models 3 and 5) as response variables are depicted in red and blue, respectively. Models with and without random effects are depicted with continuous and dashed vertical lines, respectively. Models 2–5 were fitted to all datasets. Model 1 was fitted twice, once for just the PCR dataset (black triangle) and once for all datasets (black circle). Details of these models are given in Table 2.

Our results using simulated data reveal that the 95% confidence intervals from the logistic regressions, both with and without random effects, were typically narrower than the 95% credible intervals from the proposed model (Fig. 2), often missing the true regression parameters, even when these effects were large. In contrast to these results, the 95% credible interval generated by the proposed model fitted to all datasets always included the true regression parameters. One parameter of particular importance is the intercept as it reveals the infection prevalence for individuals with mean covariate values. Our results show that all logistic regressions grossly overestimated this parameter. The simulated data also revealed that fitting the proposed model to all datasets (microscopy and PCR results from the ACD, PCD, and AACD datasets) resulted in sharper inference, both in terms of smaller bias and uncertainty, when compared to results from the proposed model fitted just to PCR results (black circle vs. black triangle, Fig. 2). This improved inference arises not only because of the larger sample size but also because the ACD and PCD datasets are more time continuous, resulting in greater variability for several covariates.

**Figure 3:**
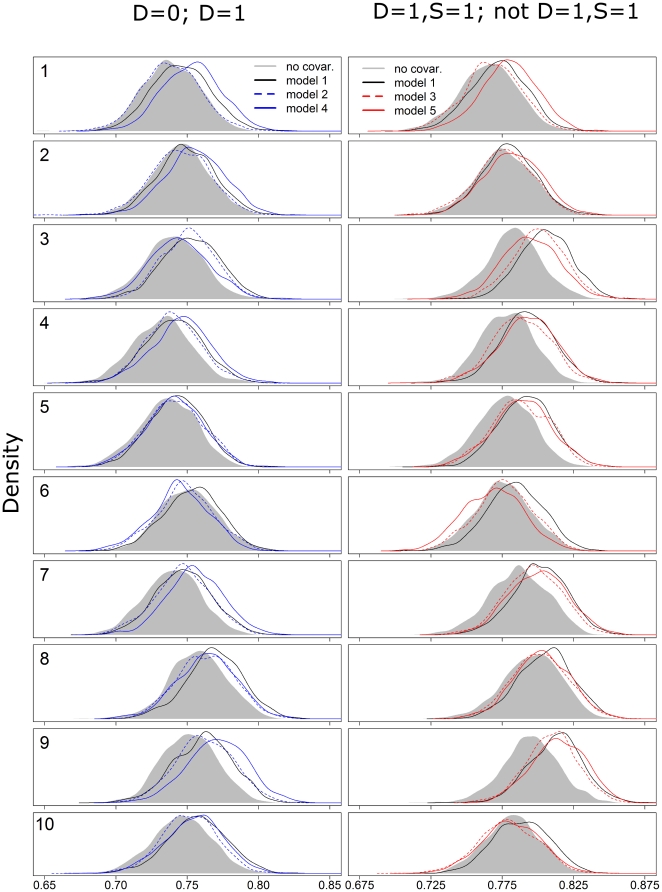
Comparison of models by out-of-sample prediction. These figures show the proportion of individuals correctly classified by each model. Numbers on the left refer to the different validation datasets. Logistic regression models with disease (models 2 and 4) and infection (models 3 and 5) as response variables are depicted in red and blue, respectively. Models with and without random effects are depicted with continuous and dashed lines, respectively. Details of these models are given in Table 2.

An important concern related to the proposed model is that it might be over-fitting the data, given that it includes almost twice as many parameters as the logistic regressions (30 vs. 17, respectively, after excluding random effects and their variances), potentially resulting in poor out-of-sample predictive ability. However, our validation results using the real data show that the proposed model had a similar or better predictive ability when compared to the logistic regression model with random effects (Fig. 3). Interestingly, even the model without any covariates had a good predictive ability, sometimes yielding equivalent or better predictions than the logistic models, with or without random effects. In contrast, the proposed model always yielded better predictions than the model without any covariates. Furthermore, the proposed model is capable of generating all predictions depicted in Fig. 3 whereas distinct logistic regressions were fit to predict these different outcomes.

### Findings from the Western Brazilian Amazon region

**Figure 4:**
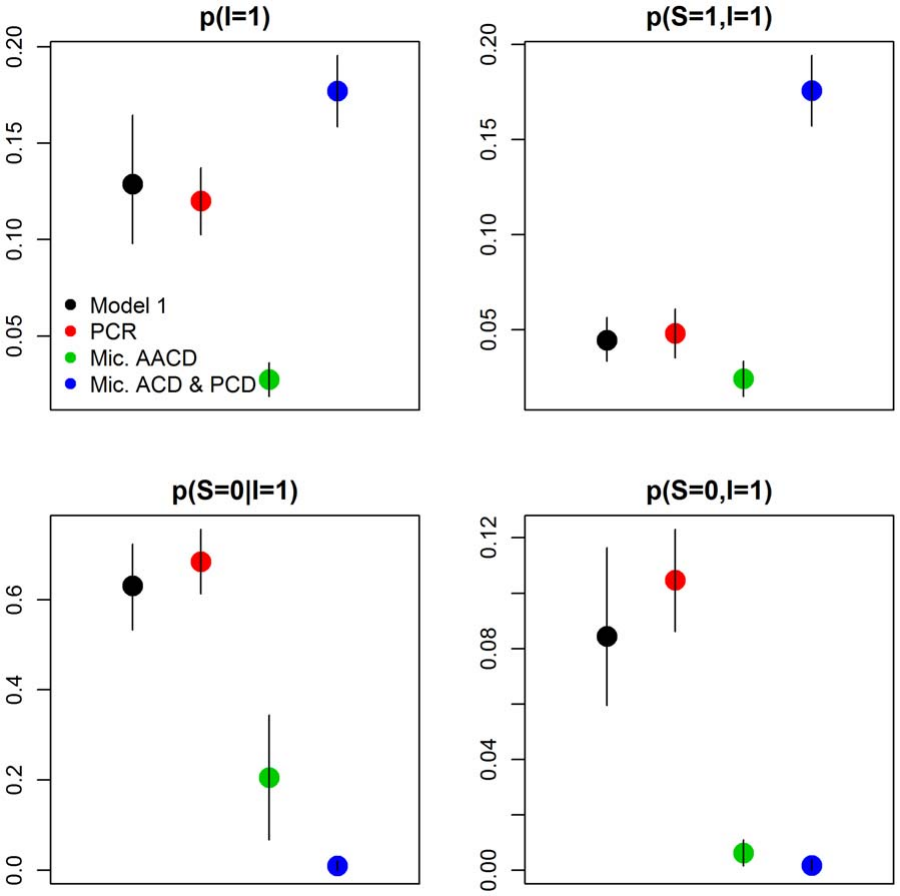
Comparison of summary statistics calculated directly from the data and generated by the proposed model. The summary statistics are infection (i.e., 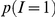) and malaria prevalence (i.e., 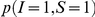), proportion of asymptomatic individuals among the pool of infected individuals (i.e., 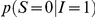) and overall proportion of asymptomatic carriers in the population (i.e., 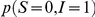). Estimates from the proposed model are depicted in black. Estimates calculated directly from the data are depicted in red (PCR data), green (microscopy results from AACD), and blue (microscopy results from ACD and PCD). Vertical lines depict 95% credible intervals for model 1 and approximate 95% confidence intervals for the other estimates, calculated as 
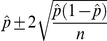
.

We estimated that the infection prevalence for the cohort we studied was approximately 0.13 (95% credible interval (CI) 0.10–0.16). Malaria prevalence was considerably lower (0.04, 95% CI 0.03–0.06) because not all individuals exhibit symptoms. From the pool of infected individuals, more than half will typically be asymptomatic (0.63, 95% CI 0.53–0.72) but the overall prevalence of asymptomatic carriers is low (0.08, 95% CI 0.06–0.12). We can compare these model-based estimates with estimates calculated directly from the data, if we assume that all individuals with a positive (or negative) detection result are infected (or not infected). Similar, but not identical, results were obtained using only PCR data (Fig. 4). On the other hand, considerably different summary statistics were obtained using microscopy, either from AACD or from the PCD/ACD datasets. These differences arise because microscopy is known to have limited ability to detect individuals with low parasitemia, which tend to be asymptomatic individuals, and because the PCD/ACD datasets include predominantly symptomatic individuals. One option would be to analyze just the PCR dataset collected with the AACD method, ignoring malaria risk information from the other datasets. However, as we showed with the simulated data and as suggested elsewhere [30], inference can be greatly improved when all datasets are jointly used if the model is able to adequately accommodate the inherent differences among datasets. Thus, we exploit the information on infection/disease prevalence and malaria risk factors from all datasets.

**Figure 5:**
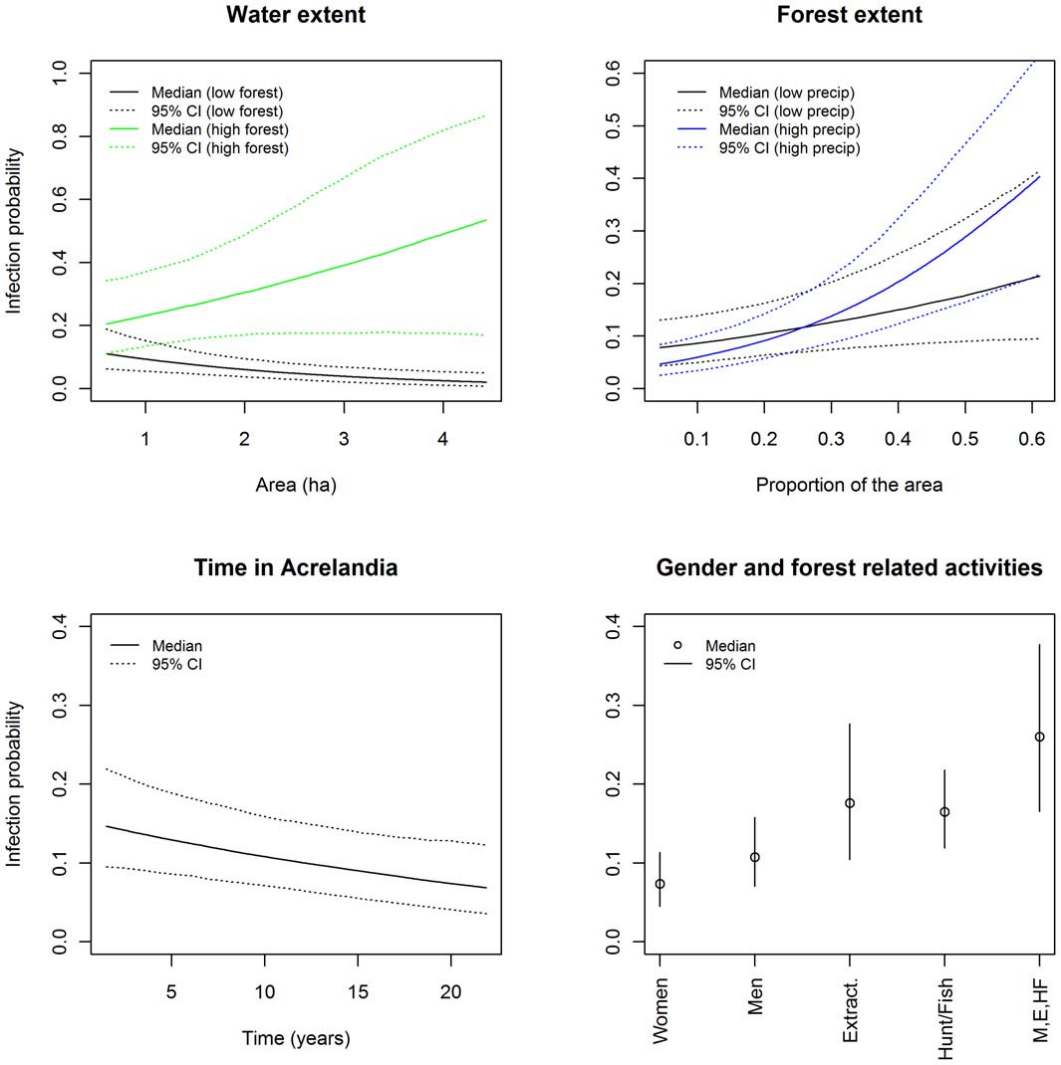
Probability of infection 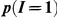 as a function of the most important covariates. The probability of infection was calculated with the other covariates fixed at their mean value. CI stands for credible interval. Lower right panel shows the independent effect of being a woman (‘Women’), being a man (‘Men’), participating on extractivism activities (‘Extract.’), and participating on hunting or fishing activities (‘Hunt/Fish’). The summed effect of being a man, partipating on extractivism and hunting/fishing activities is also shown (‘M,E,HF’).

The clearest infection risk factor was forest extent surrounding the subject's house (Table 3, Fig. 5; the marginal posterior distributions for all the estimated parameters are provided in Figure S1 and Figure S2). The effect of forest extent was further exacerbated by proximity to larger water bodies, particularly during the wet season. Furthermore, men (probably as a result of spending more time in the forest than women) and those participating in forest related activities (e.g., extractivism, hunting or fishing) were more likely to be infected (Table 3, Fig. 5). These risk factors consistently suggest that these degraded forests are prime breeding habitat for the vector. On the other hand, annual deforestation rates and working as a chain sawyer were not important risk factors. We hypothesize that the extensive use of fire for land clearing during the dry season might be responsible for this pattern. We also expected increased infection risk if the person co-inhabited a house with somebody diagnosed with malaria within the past 30 days but this was not the case, probably because infectious individuals might be diagnosed after (instead of before) the focal person is tested for malaria. Unfortunately, these past and future dependencies cannot currently be included in the model.

**Table 3:** Summary statistics for the estimated parameters.

Class	Parameter	Percentile		
		2.50%	50%	97.50%
Infection risk factors (odds-ratio)	Intercept	0.076	0.120	0.187
	Gender	0.430	0.657	0.986
	Age	0.878	1.096	1.345
	Education	0.841	1.008	1.215
	Time in Acrelandia	0.596	0.763	0.956
	Chain Sawyer	0.009	0.363	3.235
	Extractivism	1.057	1.782	2.994
	Hunting/Fishing	1.140	1.647	2.386
	Co-inhabits D^m^ = 1	0.559	0.917	1.490
	Co-inhabits D^pcr^ = 1	0.403	0.824	1.691
	Water area	0.667	0.800	0.957
	Forest area	1.430	1.923	2.569
	Annual defor.	0.719	0.909	1.139
	Monthly precip.	0.810	0.975	1.175
	Drought index	0.770	0.932	1.145
	Precip. x forest	1.004	1.183	1.405
	Drought x forest	0.803	0.958	1.155
	Water x forest	1.048	1.404	1.899
Symptoms given infection risk factors (odds-ratio)	Intercept	0.411	0.641	1.076
	Age	0.645	0.884	1.240
	Gender	0.451	0.859	1.704
	Time in Acrelandia	1.043	1.481	2.268
Other parameters (probabilities)	Mic. sensit.|S = 0	0.053	0.101	0.175
	Mic. sensit.|S = 1	0.249	0.293	0.348
	PCR Sensitivity	0.708	0.796	0.901
	PCR Specificity	0.970	0.974	0.990
	p(S = 1|I = 0)	0.015	0.023	0.034
	p(ACD|S = 1)	0.075	0.380	0.770
	p(ACD|S = 0)	0.000	0.002	0.005
	p(PCD|S = 1)	0.084	0.391	0.775
	p(PCD|S = 0)	0.000	0.001	0.002

There is some evidence that time living in Acrelandia, as a proxy for past malaria exposure, reduces the risk of being infected (Table 3, Fig. 5). This result suggests that non-naïve settlers acquire parasitological immunity and/or considerable knowledge on how to reduce one's exposure to infection. However, our results also suggest that this same factor increases the probability of feeling symptoms once infected (Table 3). One possible explanation is that non-naïve settlers are only susceptible to the more virulent *Plasmodium* strains.

**Figure 6:**
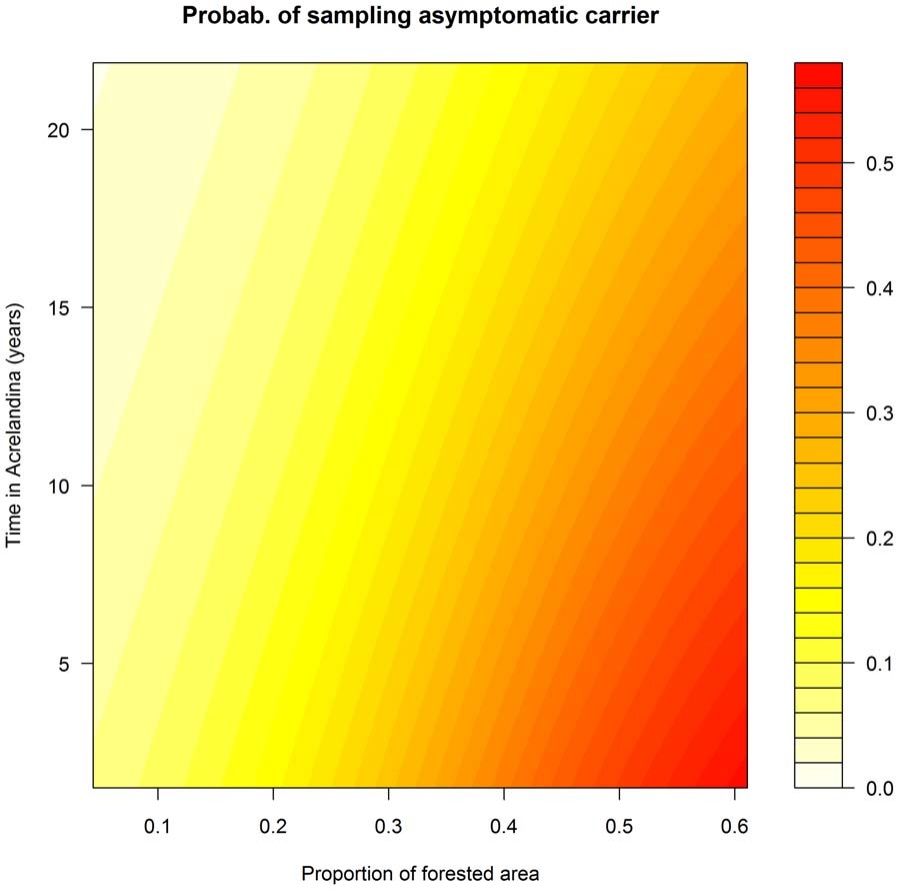
Probability of sampling an asymptomatic *Plasmodium* carrier (i.e., 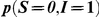). The probability of sampling an asymptomatic *Plasmodium* carrier is shown as a function of time in Acrelandia and proportion of forest area in places with abundant surface water.

Asymptomatic *Plasmodium* carriers pose a considerable public health challenge. Our results suggest ways to strategically identify these carriers. While sampling all individuals regardless of symptoms (as in AACD) might be useful, a more efficient strategy would be to sample individuals at high risk of infection but low probability of feeling symptoms given infection. In other words, we maximize 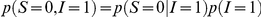 by maximizing the individual components 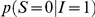 and 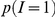. For instance, if we estimate the probability of being an asymptomatic *Plasmodium* carrier as a function of time in Acrelandia and forest extent, it becomes clear that we should preferentially sample individuals that are new to the area (thus with high 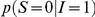) on highly forested areas with abundant surface water (thus with high 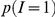) (Fig. 6).

**Figure 7:**
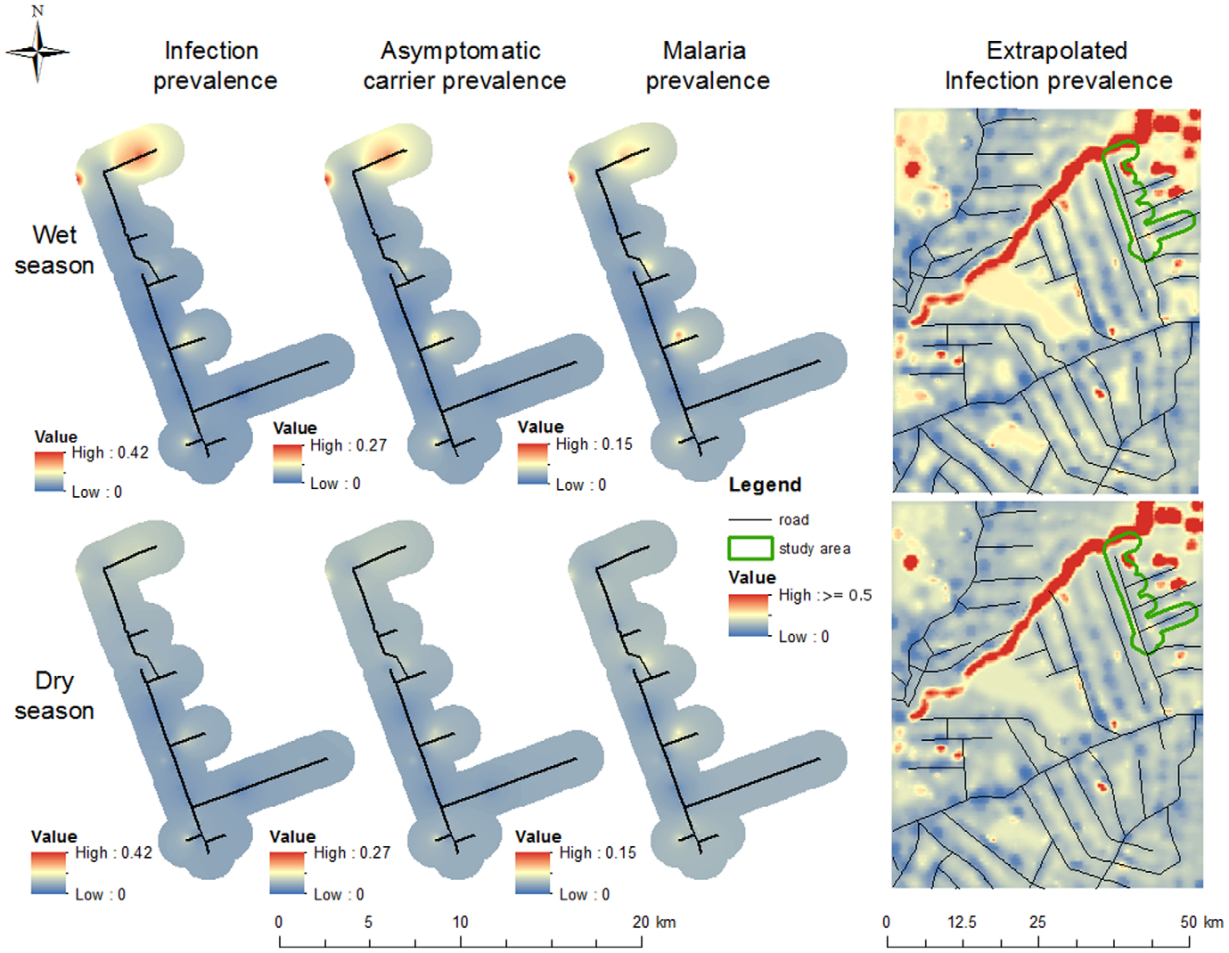
Spatial distribution of infection, asymptomatic carrier, and malaria prevalences. From left to right, maps depict interpolated surfaces of predicted infection prevalence (i.e., 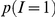), asymptomatic carrier prevalence (i.e., 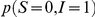)), and malaria prevalence (i.e., 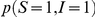), all for the studied area, and an extrapolated surface of infection prevalence. Upper and lower maps are the prevalence surfaces for the rainy and dry seasons, respectively. Interpolation was done using an inverse distance weighted algorithm.

The estimated parameters can be jointly used to make coherent predictions, relying on information from all datasets. For example, a predicted infection risk surface can be created using information on surface water and forest area (infection prevalence map in Fig. 7). These results can be extrapolated to a larger geographical region using remote sensing imagery, revealing substantial spatial heterogeneity in infection prevalence attributable to the river that crosses the upper part of the region and the large forest blocks away from the roads (extrapolated infection prevalence map in Fig. 7). These maps also highlight the striking differences in infection prevalence due to precipitation, a result greatly corroborated by recent entomological surveys conducted at the same site [31]. Besides infection risk surfaces, asymptomatic carrier risk and malaria burden surfaces can also be created, using household information on how long people have been living in Acrelandia (asymptomatic carrier and malaria prevalence maps in Fig. 7). Despite similarities, the asymptomatic carrier prevalence surface indicates that these carriers are more likely to be found in the northern part of our study area whereas infected symptomatic individuals can also be found in the central region of our study area.

As expected, we find strong influence of priors on the estimation of the PCR error rates (Fig. S2), suggesting that there was not enough information on our dataset to estimate all these parameters jointly. Microscopy sensitivity, on the other hand, was well estimated to be approximately 0.3 and 0.1, almost a three-fold difference for symptomatic and asymptomatic individuals, respectively (Table 3). Nevertheless, even for symptomatic individuals, sensitivity of microscopy was relatively low. Several quantities can be derived from these error rate estimates. For example, sampling predominantly symptomatic individuals (as is usually done in ACD/PCD) is sensible given that the probability of being infected 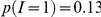 increases dramatically if the person is symptomatic 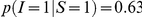. However, the challenge of using microscopy as the only method to monitor infection and disease prevalence is evident if we compare our knowledge of infection probability for symptomatic individuals before and after obtaining a negative microscopy result (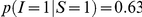 and 
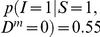
, respectively), indicating very little gain of information when microscopy yields a negative result. This finding suggests that close monitoring of individuals that are symptomatic but that have recently obtained a negative microscopy result might be warranted. On the other hand, a positive microscopy detection is very informative since 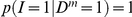. PCR results, regardless if positive or negative, were also informative since 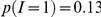 but 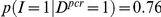 and 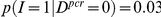.

## Discussion

Large spatial-scale patterns regarding malaria typically involves syndromic surveillance data (e.g., [32], [33]), despite limited microscopy sensitivity and the biased nature of these data. On the other hand, more reliable infection and disease prevalence estimates are often spatially and temporally restricted, relying almost exclusively on PCR data [13], [22], [34]–[36]. The proposed model uses information from both datasets to improve the estimates of infection and disease prevalence at our research site, which is then extrapolated to a larger area. Alternatively, we can infer large spatial-scale patterns of *Plasmodium* infection prevalence using the syndromic surveillance data *after* adjusting for the inherent biases in this dataset. This adjustment is only possible with the parameters estimated here and is part of our ongoing research.

Our results identify the important role of forests and forest related activities in *Plasmodium* infection risk, particularly during the rainy season and in close proximity to large water bodies (Fig. 5). Unfortunately, the data do not contain more information regarding these forests (e.g., level of forest degradation) and thus we cannot determine which characteristic of these forests are important infection risk factors. These results corroborate the findings of others that proximity to the forest enhances infection risk [22], [31], [37]–[39] but we do not find support for the idea that deforestation activity *per se* [33] or the lack of forest [40], [41] significantly increase infection risk. Our results also suggest that one of the factors most amenable to public policy is the participation in forest related activities (e.g., extractivism, hunting and fishing activities). Hunting and fishing activities are particularly popular, with nearly two thirds of the individuals in our cohort reporting that they engage in these activities. Educational campaigns might be effective in raising awareness about how participation in these activities affects one's health and the health of their family and community, particularly for those individuals more likely to exhibit symptoms given infection (i.e., non-naïve settlers).

Malaria immunity is typically portrayed as a phenomenon that depends on age (as a proxy for past malaria exposure), with severe malaria being relatively common for young children, and older cohorts having progressively less cases of severe malaria and proportionally more cases of mild malaria and asymptomatic infection [23], [42]. This descriptions refers to people exposed to malaria since birth in holoendemic countries, but it is much more complex (and less well understood) in areas with lower levels of exposure and where mild malaria predominates [23]. In these latter settings, previous studies have suggested that past exposure to malaria can decrease clinical malaria risk in rural settlers [22] and provide both anti-parasite and anti-disease immunity in traditional riverine populations [13], [34]. Our results suggest that anti-parasite immunity arises even in rural settlers. However, unlike previous studies, we find evidence that it also increases the probability of feeling symptoms once infected. We hypothesize that more experienced settlers are susceptible only to more virulent *Plasmodium* strains. Further studies are clearly needed to determine if this hypothesis is correct.

Joint models or analyzes, like ours, are models that simultaneously make inference on multiple outcomes (e.g., detection and symptom status), even allowing one outcome to influence the others (e.g., symptom status affecting detection). These models have recently become very popular in the medical statistics literature because more information and interpretability can be gained when compared to performing separate analysis of the different outcomes (e.g., [43], [44]). Another active research area in statistics focuses on the use of multiple pathogen detection methods to determine overall disease prevalence and sensitivity/specificity of these detection methods [24], [45]–[49]. A recent malaria-specific example can be found in Speybroeck et al. [50]. Our model builds on both of these trends by evaluating the risk factors of infection and symptoms given infection using data from multiple case and pathogen detection methods. Our results using simulated and real data revealed that the proposed model yields better inference on risk factors and disease/infection prevalence without over-fitting the data. To our knowledge, most of the epidemiological research regarding malaria has focused on infection risk factors. However, unlike standard logistic regression, the proposed model allows coherent inference on several other important parameters, such as detection error rates and risk factors associated with symptoms given infection. The latter is critical to advance our understanding of malaria burden and asymptomatic carriers. A direct result of this coherent inference is the identification of the need for better monitoring strategies regarding symptomatic individuals with negative microscopy results and how to sample more effectively potential asymptomatic *Plasmodium* carriers (Fig. 6). Finally, predicted surfaces of infection risk, asymptomatic carrier risk, and malaria burden allow for optimal spatial allocation of resources and malaria control activities.

One of the critical assumptions in our analysis was that data from ACD/PCD only differ from the AACD data by the unusually high proportion of symptomatic individuals. Although this is clearly a key factor, other characteristics of the individuals sampled in ACD/PCD might also be important, such as the distance of their house to the health facility. Also, our model clearly depends on having individual level data on both positive and negative microscopy tests. Unfortunately, individual level data from negative microscopy tests are typically discarded, both by the Brazilian Ministry of Health and malaria researchers, hampering future analysis of these rich datasets.

We modeled symptomatic status as a binary variable despite the fact that there is considerable variation in the type and intensity of symptoms one may exhibit [11]. Future work might allow for multinomial or continuous symptomatic status. Evidently, this would only be productive if this symptomatic status score was collected routinely in AACD *and* ACD/PCD. Another variable not included in the model is parasitemia. Precise and accurate estimates of this variable can be challenging to obtain [51]. Although new quantitative PCR methods can potentially overcome this problem, dramatic fluctuations in parasite density occur in the same individual within a short time period [18]. Therefore, the inclusion of parasitemia into an analysis like ours remains an important challenge. Furthermore, there is no way to distinguish new *Plasmodium* infections from recrudescence and relapses, even using modern genotyping technology, given that an individual might be initially infected by multiple strains and/or re-infected by the same common strain [52], [53]. Thus, what we have called infection risk factors actually refers to the risk factors of having a relapse, a recrudescence, and/or a new infection. Finally, because *P. vivax* and *P. falciparum* are particularly prevalent in the region, it would be interesting to evaluate if the probability of feeling symptoms given infection or the infection risk factors differ among these species. This remains an important research topic.

Using malaria in the Western Brazilian Amazon as a case study, we have shown that the modeling framework presented here can exploit information from multiple datasets to shed light on several aspects of an infectious disease (e.g., infection risk factors, risk factors associated with symptoms given infection, detection error rates) that are critical for its monitoring and control (e.g., indicating how to efficiently search for asymptomatic carriers and which symptomatic individuals should be closely monitored). While standard logistic regressions are undoubtedly important tools, these statistical models are not well suited to integrate multiple datasets. We believe that the Bayesian modeling framework described here fundamentally enhances our ability to overcome this challenge, being broadly applicable to other settings and diseases whenever asymptomatic carriers are an important public health concern and multiple datasets are available.

## Supporting Information

Figure S1 Red vertical lines are drawn at zero for reference. CI stands for credible interval.
(TIFF)

Figure S2The first row of graphics displays the estimated parameters associated with 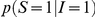. Red vertical lines are drawn at zero for reference. The second row of graphics displays the estimated detection parameters of microscopy (which depends on symptomatic status S) and PCR. The last row of graphics displays the estimated probability of feeling symptomatic given no infection (i.e., 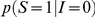) and several other estimated probabilities associated with the different case detection methods. ACD and PCD stand for active and passive case detection, respectively. CI stands for credible interval.(TIFF)

Table S1 Likelihood of each of the possible outcomes in AACD.
(DOC)

Table S2 Likelihood of each of the possible outcomes in ACD and PCD.
(DOC)

Table S3 Summary of parameter values adopted for the simulated data.
(DOC)

Table S4 Number of microscopy and PCR results for the different sampling designs, both from the original and simulated datasets.
(DOC)

Appendix S1 Description of covariates.
(DOC)

Appendix S2 Description of likelihood.
(DOC)

Appendix S3 Full conditional distribution for the parameters sampled via a Gibbs sampling step.
(DOC)

Appendix S4 Description of how data were simulated.
(DOC)
