# Supplementary figures and images for: Enhanced Understanding of Infectious Diseases by Fusing Multiple Datasets: A Case Study on Malaria in the Western Brazilian Amazon Region
Source: PLoS One. 2011 Nov 8;6(11):e27462. doi: 10.1371/journal.pone.0027462 (PMC3210805; doi:10.1371/journal.pone.0027462)

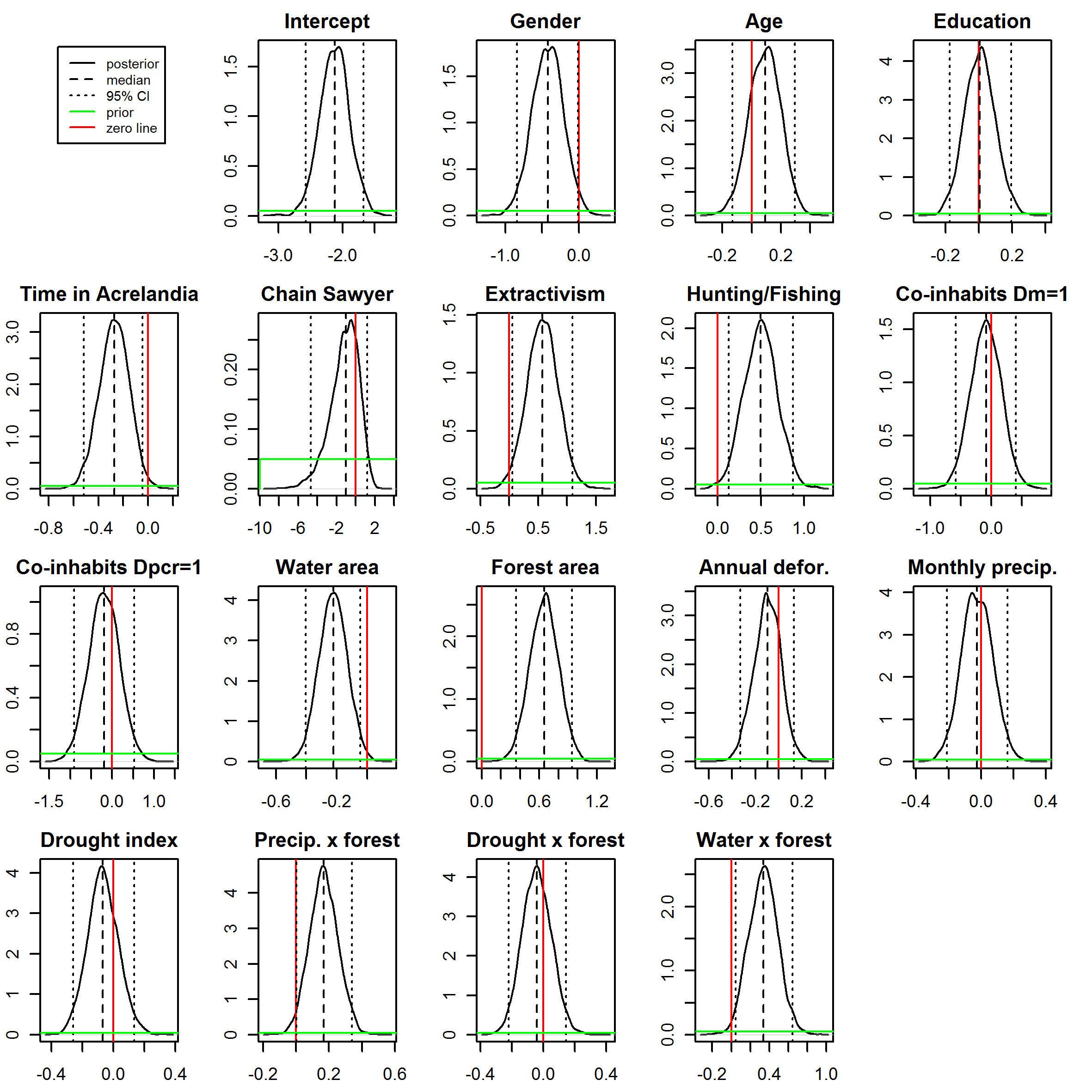

Supplement: Figure S1 — Red vertical lines are drawn at zero for reference. CI stands for credible interval. (TIFF) [file pone.0027462.s001.tiff]

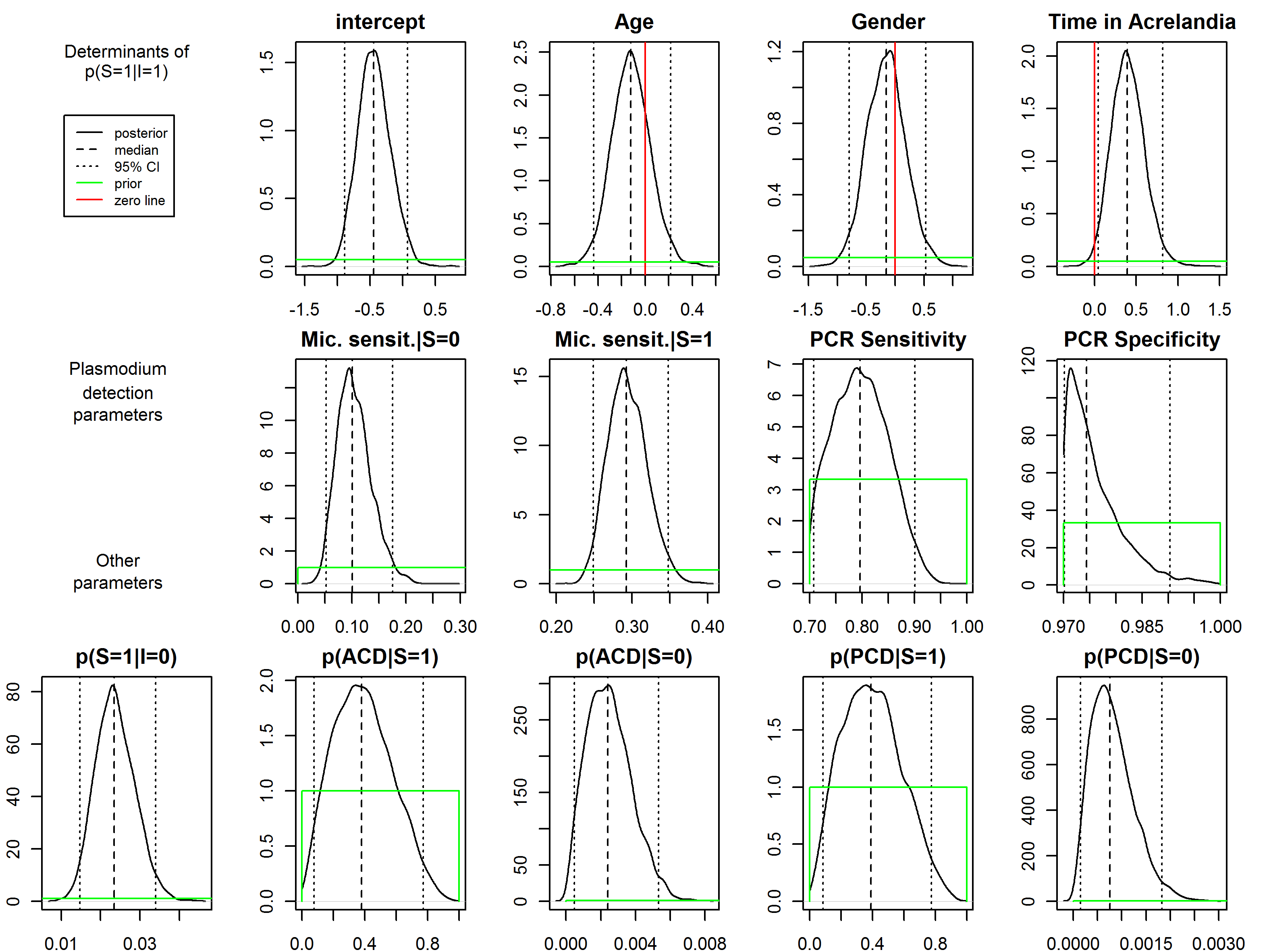

Supplement: Figure S2 — The first row of graphics displays the estimated parameters associated with . Red vertical lines are drawn at zero for reference. The second row of graphics displays the estimated detection parameters of microscopy (which depends on symptomatic status S) and PCR. The last row of graphics displays the estimated probability of feeling symptomatic given no infection (i.e., ) and several other estimated probabilities associated with the different case detection methods. ACD and PCD stand for active and passive case detection, respectively. CI stands for credible interval. (TIFF) [file pone.0027462.s002.tiff]
