# Supplementary material for: Enhanced Understanding of Infectious Diseases by Fusing Multiple Datasets: A Case Study on Malaria in the Western Brazilian Amazon Region
Source: PLoS One. 2011 Nov 8;6(11):e27462. doi: 10.1371/journal.pone.0027462 (PMC3210805; doi:10.1371/journal.pone.0027462)
Supplement: Table S1 — Likelihood of each of the possible outcomes in AACD. (DOC) [file pone.0027462.s003.doc]

| $D^{pcr}$ | $D^{m}$ | S | Likelihood |
| --- | --- | --- | --- |
| X | 0 | X | $[(1-\alpha_{1})B+(1-\alpha_{0})(1-B)]A+(1-A)$ |
| X | 1 | X | $[\alpha_{1}B+\alpha_{0}(1-B)]A$ |
| 0 | X | X | $(1-\delta)A+\pi(1-A)$ |
| 1 | X | X | $\delta A+(1-\pi)(1-A)$ |
| X | 1 | 0 | $\alpha_{0}(1-B)A$ |
| X | 0 | 0 | $(1-\alpha_{0})(1-B)A+(1-C)(1-A)$ |
| X | 0 | 1 | $(1-\alpha_{1})BA+C(1-A)$ |
| X | 1 | 1 | $\alpha_{1}BA$ |
| 1 | X | 0 | $\delta(1-B)A+(1-\pi)(1-C)(1-A)$ |
| 0 | X | 0 | $(1-\delta)(1-B)A+\pi(1-C)(1-A)$ |
| 0 | X | 1 | $(1-\delta)BA+\pi C(1-A)$ |
| 1 | X | 1 | $\delta BA+(1-\pi)C(1-A)$ |
| 1 | 0 | X | $\delta[(1-\alpha_{1})B+(1-\alpha_{0})(1-B)]A+(1-\pi)(1-A)$ |
| 0 | 0 | X | $(1-\delta)[(1-\alpha_{1})B+(1-\alpha_{0})(1-B)]A+\pi(1-A)$ |
| 0 | 1 | X | $(1-\delta)[\alpha_{1}B+\alpha_{0}(1-B)]A$ |
| 1 | 1 | X | $\delta[\alpha_{1}B+\alpha_{0}(1-B)]A$ |
| 0 | 0 | 0 | $(1-\delta)(1-\alpha_{0})(1-B)A+\pi(1-C)(1-A)$ |
| 1 | 1 | 1 | $\delta\alpha_{1}BA$ |
| 0 | 0 | 1 | $(1-\delta)(1-\alpha_{1})BA+\pi C(1-A)$ |
| 0 | 1 | 0 | $(1-\delta)\alpha_{0}(1-B)A$ |
| 1 | 0 | 0 | $\delta(1-\alpha_{0})(1-B)A+(1-\pi)(1-C)(1-A)$ |
| 1 | 1 | 0 | $\delta\alpha_{0}(1-B)A$ |
| 0 | 1 | 1 | $(1-\delta)\alpha_{1}BA$ |
| 1 | 0 | 1 | $\delta(1-\alpha_{1})BA+(1-\pi)C(1-A)$ |

Note: ‘X’ denotes missing data.
