# Supplementary material for: Enhanced Understanding of Infectious Diseases by Fusing Multiple Datasets: A Case Study on Malaria in the Western Brazilian Amazon Region
Source: PLoS One. 2011 Nov 8;6(11):e27462. doi: 10.1371/journal.pone.0027462 (PMC3210805; doi:10.1371/journal.pone.0027462)
Supplement: Table S2 — Likelihood of each of the possible outcomes in ACD and PCD. (DOC) [file pone.0027462.s004.doc]

| $D^{pcr}$ | $D^{m}$ | *S* | Likelihood |
| --- | --- | --- | --- |
| X | 0 | X | $\frac{[(1-\alpha_{1})BE+(1-\alpha_{0})(1-B)F]A+[CE+(1-C)F](1-A)}{E(BA+C(1-A))+F[1-(BA+C(1-A))]}$ |
| X | 1 | X | $\frac{[\alpha_{1}BE+\alpha_{0}(1-B)F]A}{E(BA+C(1-A))+F[1-(BA+C(1-A))]}$ |
| X | 1 | 0 | $\frac{\alpha_{0}(1-B)FA}{E(BA+C(1-A))+F[1-(BA+C(1-A))]}$ |
| X | 0 | 0 | $\frac{F[(1-\alpha_{0})(1-B)A+(1-C)(1-A)]}{E(BA+C(1-A))+F[1-(BA+C(1-A))]}$ |
| X | 0 | 1 | $\frac{E[(1-\alpha_{1})BA+C(1-A)]}{E(BA+C(1-A))+F[1-(BA+C(1-A))]}$ |
| X | 1 | 1 | $\frac{\alpha_{1}BEA}{E(BA+C(1-A))+F[1-(BA+C(1-A))]}$ |

Note: ‘X’ denotes missing data.
