# Supplementary material for: Enhanced Understanding of Infectious Diseases by Fusing Multiple Datasets: A Case Study on Malaria in the Western Brazilian Amazon Region
Source: PLoS One. 2011 Nov 8;6(11):e27462. doi: 10.1371/journal.pone.0027462 (PMC3210805; doi:10.1371/journal.pone.0027462)
Supplement: Table S3 — Summary of parameter values adopted for the simulated data. (DOC) [file pone.0027462.s005.doc]

| Parameter | Description | Values |
| --- | --- | --- |
| $\alpha_{1}$ | Microscopy sensitivity given S=1 | 0.6 |
| $\alpha_{0}$ | Microscopy sensitivity given S=0 | 0.4 |
| $\delta$ | PCR sensitivity | 0.8 |
| $\pi$ | PCR specificity | 0.985 |
| $\beta$ | Covariates of infection risk factors | One of the following: -0.2, -0.1, 0, 0.1, and 0.2 |
| $\sigma_{ind}$ | Standard deviation of the individual level random effects | 0.25 |
| $\sigma_{h}$ | Standard deviation of the household level random effects | 0.5 |
| $\gamma$ | Covariates of risk factors of symptoms given infection | One of the following: 2, -0.5, 0, and 0.1 |
| $p(S=1\vert I=0)$ | Probability of symptoms given no infection | 0.03 |
|  | Probability of being sampled through PCD given no symptoms | 0.012 |
|  | Probability of being sampled through PCD given symptoms | 0.6 |
| $p(ACD\vert S=0)$ | Probability of being sampled through ACD given no symptoms | 0.02 |
| $p(ACD\vert S=1)$ | Probability of being sampled through ACD given symptoms | 0.3 |
