# Supplementary material for: Enhanced Understanding of Infectious Diseases by Fusing Multiple Datasets: A Case Study on Malaria in the Western Brazilian Amazon Region
Source: PLoS One. 2011 Nov 8;6(11):e27462. doi: 10.1371/journal.pone.0027462 (PMC3210805; doi:10.1371/journal.pone.0027462)
Supplement: Table S4 — Number of microscopy and PCR results for the different sampling designs, both from the original and simulated datasets. (DOC) [file pone.0027462.s006.doc]

|  | Original dataset | | Simulated dataset | |
| --- | --- | --- | --- | --- |
| Sampling design | PCR | Microscopy | PCR | Microscopy |
| AACD | 1400 | 1383 | 1291 | 1291 |
| ACD | 0 | 940 | 0 | 773 |
| PCD | 0 | 754 | 0 | 1294 |
