# Supplementary material for: Enhanced Understanding of Infectious Diseases by Fusing Multiple Datasets: A Case Study on Malaria in the Western Brazilian Amazon Region
Source: PLoS One. 2011 Nov 8;6(11):e27462. doi: 10.1371/journal.pone.0027462 (PMC3210805; doi:10.1371/journal.pone.0027462)
Supplement: Appendix S1 — Description of covariates. (DOC) [file pone.0027462.s007.doc]

Appendix S1 – Description of covariates

Whenever linking secondary environmental data to malaria data, it is crucial to determine the area surrounding each household that can be reasonably expected to influence the observed *Plasmodium* infection risk (onwards ‘area of influence’). For our study, we set the area of influence for each household as a circle with radius of 1,000m centered at the household. We chose this radius because if reflects the area where we expect people to spend most of their time and because the malaria vector is known to have a flight range on this order of magnitude .

The covariates for infection risk were:

- *gender* (binary, women=1 and men=0);
- *chain sawyer* (binary, yes=1 and no=0): if works primarily as a chain sawyer;
- *age* (in years);
- *education* (in years of schooling);
- *time in Acrelandia* (in years): Clinical and parasitological immunity is often higher with greater past exposure to malaria . Because people in rural land settlements often come from malaria-free regions , we added the time living in Acrelandia as a proxy for past exposure to malaria;
- *extractivism* (binary, yes=1 and no=0)*:* if participates in extractivism activities;
- *hunt/fish* (binary, yes=1 and no=0): if participates in hunting or fishing activities;
- *co-inhabits D^m^=1* (binary, yes=1 and no=0): if the person being tested shares their house with somebody that had a positive microscopy result in the past 30 days;
- *co-inhabits D^pcr^=1* (binary, yes=1 and no=0): if the person being tested shares their house with somebody that had a positive PCR result in the past 30 days;
- *water* *area* (surface water area in 2008, in ha): this was estimated by visual interpretation of high-spatial resolution imagery (2 x 2m and 8 x 8m pixels for panchromatic and multispectral images, respectively), acquired by FORMOSAT in 2008. Many of these water bodies can be considered permanent landscape features (e.g., natural rivers, ponds created to raise fish or provide water to the cattle). Thus, in the absence of high-spatial resolution imagery from earlier years, we assumed that these water bodies were probably present throughout 2004-2008.
- *forest area* (in ha in 2004): the Brazilian Space Agency (INPE) provides yearly land cover classification maps for the entire Brazilian Amazon based on a semi-automated analysis of Landsat imagery . Using these maps, we were able to determine forest extent in 2004 within the area of influence of each household;
- *deforestation rate* (in ha yr^-1^): using data from INPE (described above), we determined the yearly deforested area within the area of influence of each household;
- *precipitation* (monthly average, in mm hour^-1^): Precipitation data came from the Tropical Rainfall Measuring Mission - TRMM . We used the ‘3B43 Monthly 0.25 x 0.25 degree merged TRMM and other sources estimates’ product, with a one month time lag. The assumption regarding this time lag is that water affects the vector mainly through its breeding habitat. Therefore, changes in precipitation should only affect infection risk on the following month since this is the minimum necessary time for the larvae to become an adult, the adult to be infected and finally the adult to become infectious.
- *drought index* (monthly, in mm)*:* this drought index is determined by an algorithm that takes into account precipitation and evapotranspiration to calculate water deficit. The details of this algorithm are given in Aragao *et al.* . Despite its simplicity, this index has been used extensively to characterize drought in the region . Precipitation data came from TRMM and we used a one month time lag with the same rationale as for precipitation.

We also added interaction terms involving precipitation/forest area, water deficit/forest area and water area/forest area. All continuous covariates were standardized by subtracting their mean and dividing by their standard deviation. None of the covariates listed above were highly correlated (i.e., |r|<0.8)

References

1. Charlwood JD (1996) Biological variation in Anopheles darlingi Root. Mem Inst Oswaldo Cruz 91: 391-398.

2. Castro CS, Sawyer DO, Singer BH (2007) Spatial patterns of malaria in the Amazon: implications for surveillance and targeted interventions. Health & Place 13: 368-380.

3. Keiser J, Singer BH, Utzinger J (2005) Reducing the burden of malaria in different eco-epidemiological settings with environmental management: a systematic review. Lancet Infectious Diseases 5: 695-708.

4. Aguas R, White LJ, Snow RW, Gomes MGM (2008) Prospects for malaria eradication in Sub-Saharan Africa. PLosS ONE 3: e1767.

5. Coimbra CEA (1988) Human factors in the epidemiology of malaria in the Brazilian Amazon. Human Organization 47: 254 - 260

6. da Silva-Nunes M, Codeco CT, Malafronte RS, Silva NS, Juncansen C, et al. (2008) Malaria on the Amazonian frontier: transmission dynamics, risk factors, spatial distribution, and prospects for control. Am J Trop Med Hyg 79: 624-635.

7. INPE (2011) Projeto Prodes: monitoramento da floresta amazonica brasileira por satelite. Sao Jose dos Campos, SP, Brasil.

8. NASA (2010) 3B43: Montly 0.25 x 0.25 degree merged TRMM and other sources estimates. Maryland, USA.: NASA Distrib. Active Arch. Cent., Goddard Space Flight Cent. Earth Sci.

9. Aragao LEOC, Malhi Y, Roman-Cuesta RM, Saatchi S, Anderson LO, et al. (2007) Spatial patterns and fire response of recent Amazonian droughts. Geophysical Research Letters 34. doi: 10.1029/2006GL028946

10. Phillips OL, Aragao LEOC, Lewis SL, Fisher JB, Lloyd J, et al. (2009) Drought sensitivity of the Amazon rainforest. Science 323: 1344-1347.

11. Lewis SL, Brando PM, Phillips OL, van der Heijden GMF, Nepstad D (2011) The 2010 Amazon drought. Science 331: 554.
