# Supplementary material for: Enhanced Understanding of Infectious Diseases by Fusing Multiple Datasets: A Case Study on Malaria in the Western Brazilian Amazon Region
Source: PLoS One. 2011 Nov 8;6(11):e27462. doi: 10.1371/journal.pone.0027462 (PMC3210805; doi:10.1371/journal.pone.0027462)
Supplement: Appendix S2 — Description of likelihood. (DOC) [file pone.0027462.s008.doc]

Appendix S2 – Description of likelihood

We first show how the likelihoods were derived and then we provide the formulae for them.

*Derivation of likelihood:*

When only microscopy results are available for AACD, the likelihood is:

$$p(D^{m})=p(D^{m}|I=1)p(I=1)+p(D^{m}|I=0)p(I=0)$$

$=[p(D^{m}|S=1,I=1)p(S=1|I=1)+p(D^{m}|S=0,I=1)p(S=0|I=1)]p(I=1)+p(D^{m}|I=0)p(I=0)$ When microscopy results and symptom statuses are available for AACD, the likelihood is:

$$p(D^{m},S)=p(D^{m},S|I=1)p(I=1)+p(D^{m},S|I=0)p(I=0)$$

$$=p(D^{m}|S,I=1)p(S|I=1)p(I=1)+p(D^{m}|I=0)p(S|I=0)p(I=0)$$

When only PCR results are available for AACD, the likelihood is:

$$p(D^{pcr})=p(D^{pcr}|I=1)p(I=1)+p(D^{pcr}|I=0)p(I=0)$$

When PCR results and symptom statuses are available for AACD, the likelihood is:

$$p(D^{pcr},S)=p(D^{pcr},S|I=1)p(I=1)+p(D^{pcr},S|I=0)p(I=0)$$

$$=p(D^{pcr}|I=1)p(S|I=1)p(I=1)+p(D^{pcr}|I=0)p(S|I=0)p(I=0)$$

When PCR and microscopy results are available for AACD, the likelihood is:

$$p(D^{pcr},D^{m})=p(D^{pcr},D^{m}|I=1)p(I=1)+p(D^{pcr},D^{m}|I=0)p(I=0)$$

$$=p(D^{pcr}|I=1)p(D^{m}|I=1)p(I=1)+p(D^{pcr}|I=0)p(D^{m}|I=0)p(I=0)$$

$$\begin{matrix} =p(D^{pcr}|I=1)[p(D^{m}|S=1,I=1)p(S=1|I=1)+p(D^{m}|S=0,I=1)p(S=0|I=1)]p(I=1)+ \\ p(D^{pcr}|I=0)p(D^{m}|I=0)p(I=0) \end{matrix}$$

When symptom statuses, PCR and microscopy results are available for AACD, the likelihood is:

$$p(D^{pcr},D^{m},S)=p(D^{pcr},D^{m},S|I=1)p(I=1)+p(D^{pcr},D^{m},S|I=0)p(I=0)$$

$$=p(D^{pcr},D^{m}|S,I=1)p(S|I=1)p(I=1)+p(D^{pcr},D^{m}|S,I=0)p(S|I=0)p(I=0)$$

$$=p(D^{pcr}|I=1)p(D^{m}|S,I=1)p(S|I=1)p(I=1)+p(D^{pcr}|I=0)p(D^{m}|S,I=0)p(S|I=0)p(I=0)$$

When only microscopy results are available for ACD (or PCD, if we substitute ACD for PCD in the formulae below), the likelihood is:

$$p(D^{m}|ACD)=p(D^{m}|S=1,ACD)p(S=1|ACD)+p(D^{m}|S=0,ACD)p(S=0|ACD)$$

When microscopy results and symptom statuses are available for ACD (or PCD, if we substitute ACD for PCD in the formulae below), the likelihood is:

$$p(D^{m},S|ACD)=p(D^{m},S|I=1,ACD)p(I=1|ACD)+p(D^{m},S|I=0,ACD)p(I=0|ACD)$$

$$=p(D^{m}|S,I=1,ACD)p(S|I=1,ACD)p(I=1|ACD)+p(D^{m}|S,I=0,ACD)p(S|I=0,ACD)p(I=0|ACD)$$

$$=p(D^{m}|S,I=1)\frac{p(I=1|S,ACD)p(S,ACD)}{p(I=1,ACD)}p(I=1|ACD)+p(D^{m}|S,I=0)\frac{p(I=0|S,ACD)p(S,ACD)}{p(I=0,ACD)}p(I=0|ACD)$$

$$=p(D^{m}|S,I=1)p(I=1|S,ACD)p(S|ACD)+p(D^{m}|S,I=0)p(I=0|S,ACD)p(S|ACD)$$

$$=[p(D^{m}|S,I=1)p(I=1|S)+p(D^{m}|S,I=0)p(I=0|S)]\frac{p(ACD|S)p(S)}{p(ACD)}$$

$$=[p(D^{m}|S,I=1)p(S|I=1)p(I=1)+p(D^{m}|S,I=0)p(S|I=0)p(I=0)]\frac{p(ACD|S)}{p(ACD)}$$

*Likelihood formulae:*

To avoid clutter in our equations, we denote $A=p(I_{i,t}=1)=\frac{1}{1+e^{-(X_{i,t}\beta+\phi_{i}+\vartheta_{h[i]})}}$, $B=p(S_{i,t}=1|I_{i,t}=1)=\frac{1}{1+e^{-(\gamma_{i,t}y)}}$, and $C=p(S_{i,t}=1|I_{i,t}=0)$. For ACD, *E* and *F* denote $p(ACD|S_{i,t}=1)$ and , respectively. For PCD, *E* and *F* denote and , respectively. We chose to suppress subscripts but it is understood that everything is for a given individual *i* residing at household *h* at time *t*. The likelihood formulae using the above notation are given in Table S1 and S2.
