# Supplementary material for: Enhanced Understanding of Infectious Diseases by Fusing Multiple Datasets: A Case Study on Malaria in the Western Brazilian Amazon Region
Source: PLoS One. 2011 Nov 8;6(11):e27462. doi: 10.1371/journal.pone.0027462 (PMC3210805; doi:10.1371/journal.pone.0027462)
Supplement: Appendix S3 — Full conditional distribution for the parameters sampled via a Gibbs sampling step. (DOC) [file pone.0027462.s009.doc]

Appendix S3 –full conditional distribution for the parameters sampled via a gibbs sampling step

The parameters that were updated via a Gibbs sampling step were the variances for the individual and household-level random effects. The full conditional distributions for these parameters are given below:

$$\frac{1}{\sigma_{ind}^{2}}\sim Gamma(\frac{N_{ind}-1}{2},\frac{\sum_{i=1}^{N_{ind}} \phi_{i}}{2})I(0<\sigma_{ind}<100)$$

$$\frac{1}{\sigma_{h}^{2}}\sim Gamma(\frac{N_{h}-1}{2},\frac{\sum_{h=1}^{N_{h}} \vartheta_{h}}{2})I(0<\sigma_{h}<100)$$

where and are individual and household random effects, respectively, and and are the total number of individuals and households, respectively.
