# Supplementary material for: Enhanced Understanding of Infectious Diseases by Fusing Multiple Datasets: A Case Study on Malaria in the Western Brazilian Amazon Region
Source: PLoS One. 2011 Nov 8;6(11):e27462. doi: 10.1371/journal.pone.0027462 (PMC3210805; doi:10.1371/journal.pone.0027462)
Supplement: Appendix S4 — Description of how data were simulated. (DOC) [file pone.0027462.s010.doc]

Appendix S4 – Description of how data were simulated

To make a realistic comparison of the different methods, we tried to mimic the original dataset as closely as possible. Therefore, we used the same covariate values as in the original dataset to simulate the data. To evaluate how reliably each method estimated effects of different sizes (as well as no effect at all), we assigned one of the following values to the risk factor parameters, both of infection and symptoms given infection: -0.5, -0.2, -0.1, 0, 0.1, and 0.2. All the remaining parameters were assigned values close to what we had already estimated in previous runs of our model. All these parameter values are summarized in Table S3.

The simulated dataset had approximately the same number of microscopy and PCR results for the different sampling designs (AACD, PCD, and ACD) as the original dataset (Table S4).
